# Supplementary figures and images for: Forward genetic screen in zebrafish identifies new fungal regulators that limit host-protective Candida-innate immune interaction
Source: mBio. 2025 Apr 2;16(5):e00529-25. doi: 10.1128/mbio.00529-25 (PMC12077120; doi:10.1128/mbio.00529-25)

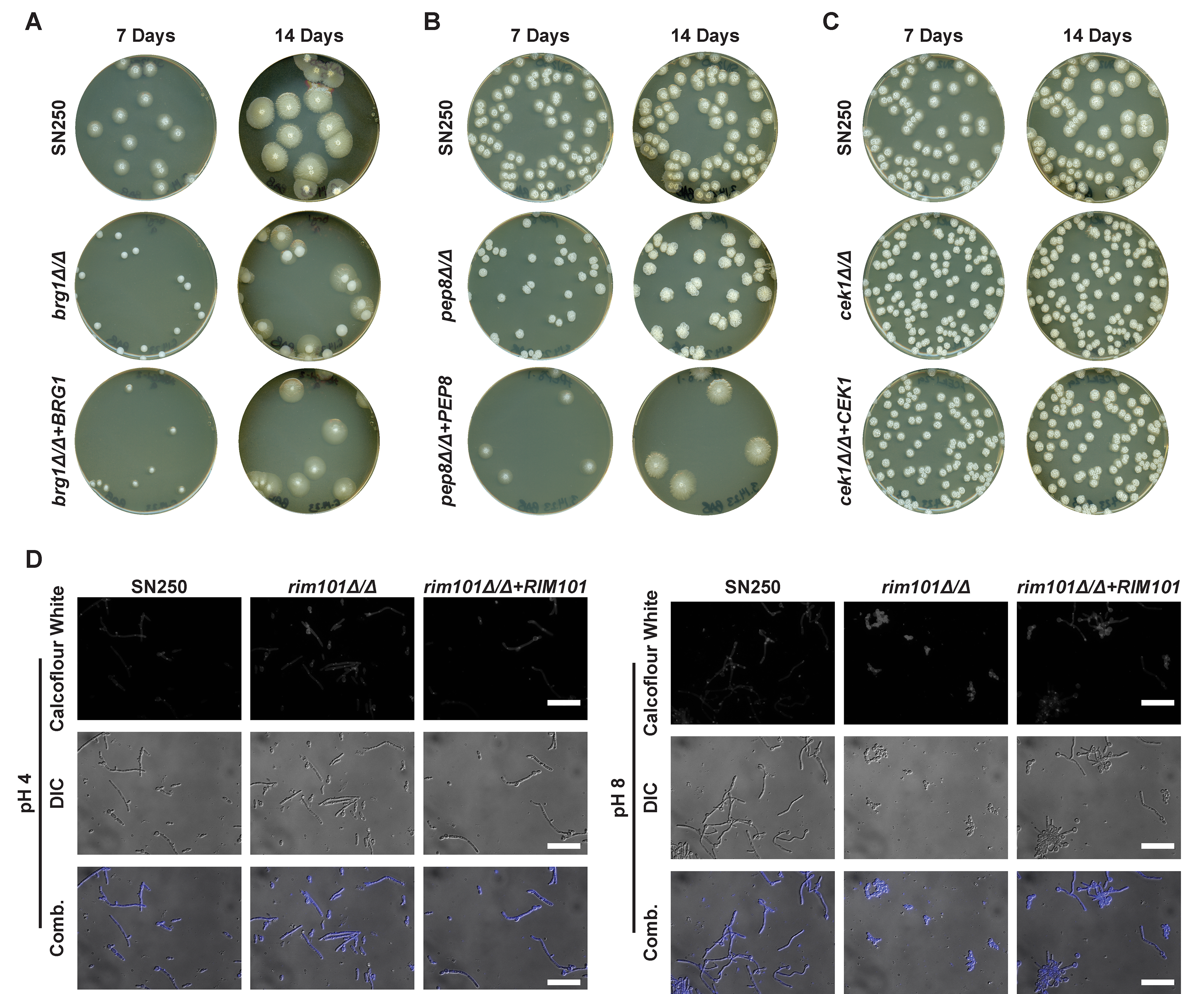

Supplement: Fig. S1 — Complementation partially restores in vitro phenotypes of brg1∆/∆, pep8∆/∆, cek1∆/∆, and rim101∆/∆ mutants. [file mbio.00529-25-s0002.tif]

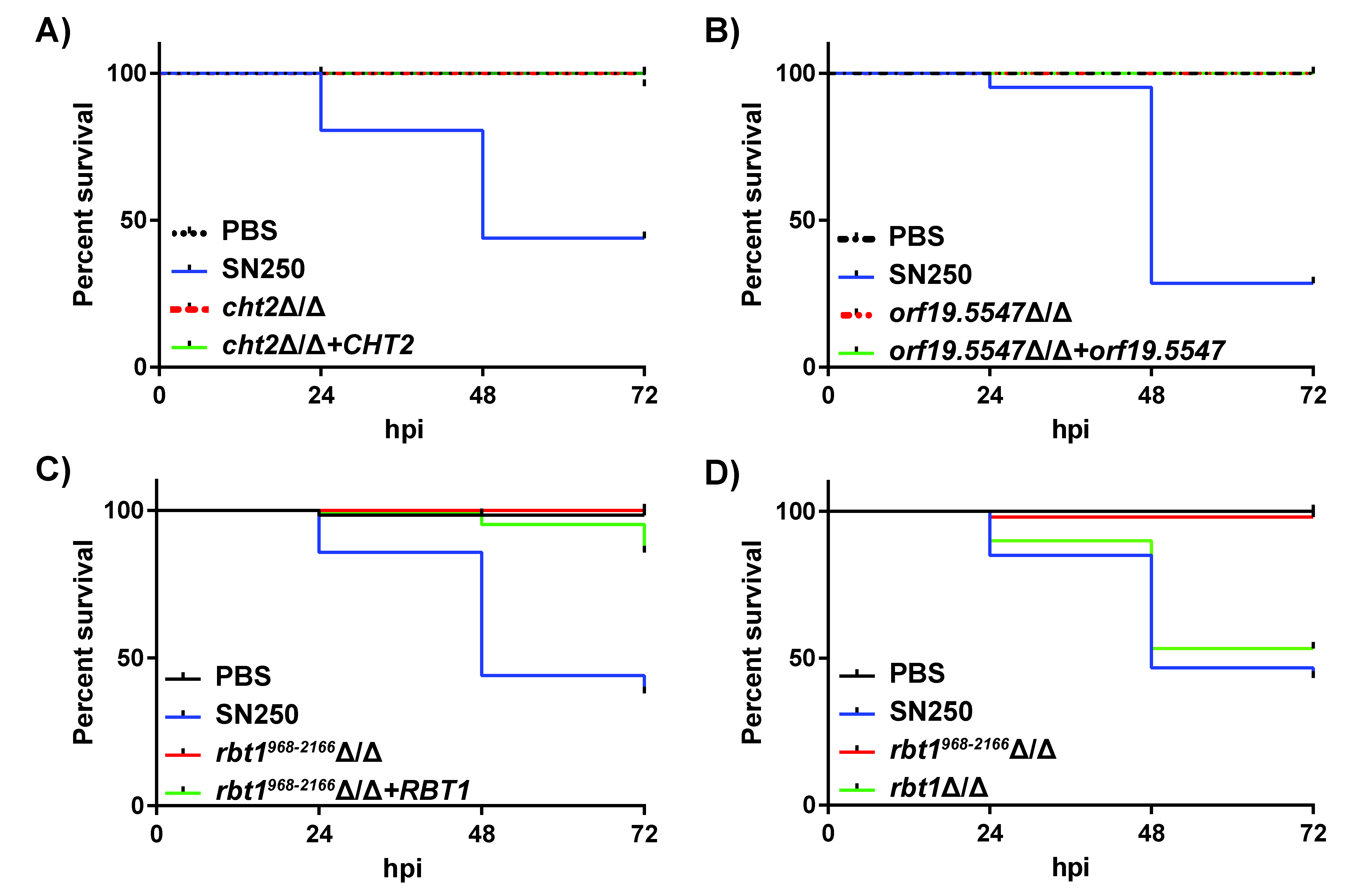

Supplement: Fig. S2 — Complementation did not restore the virulence of cht2∆/∆, orf19.5547∆/∆, or rbt1968-2166∆/∆. [file mbio.00529-25-s0003.tif]

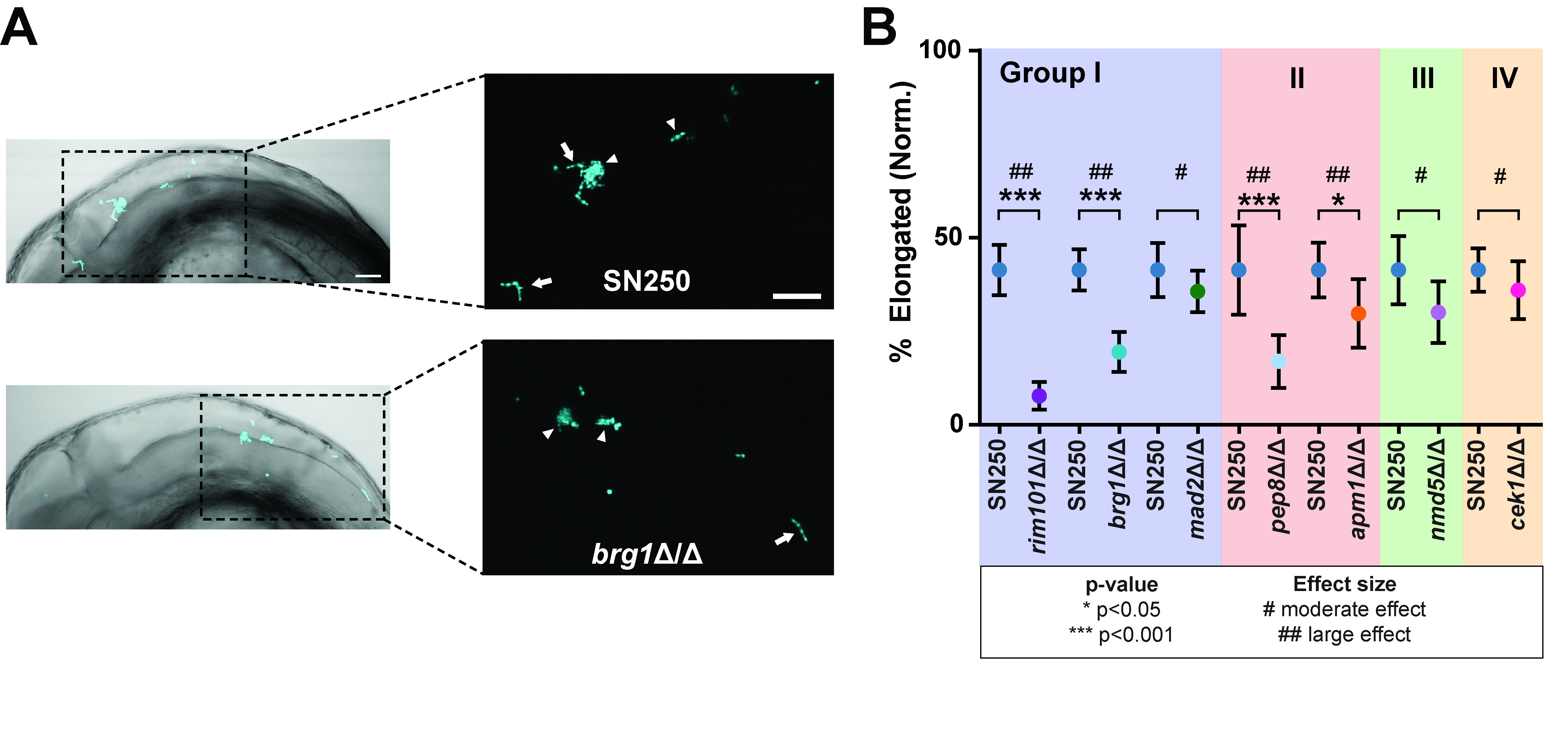

Supplement: Fig. S3 — Some mutants show fewer elongated cells in the zebrafish hindbrain at 4-6 hours post infection. [file mbio.00529-25-s0004.tif]

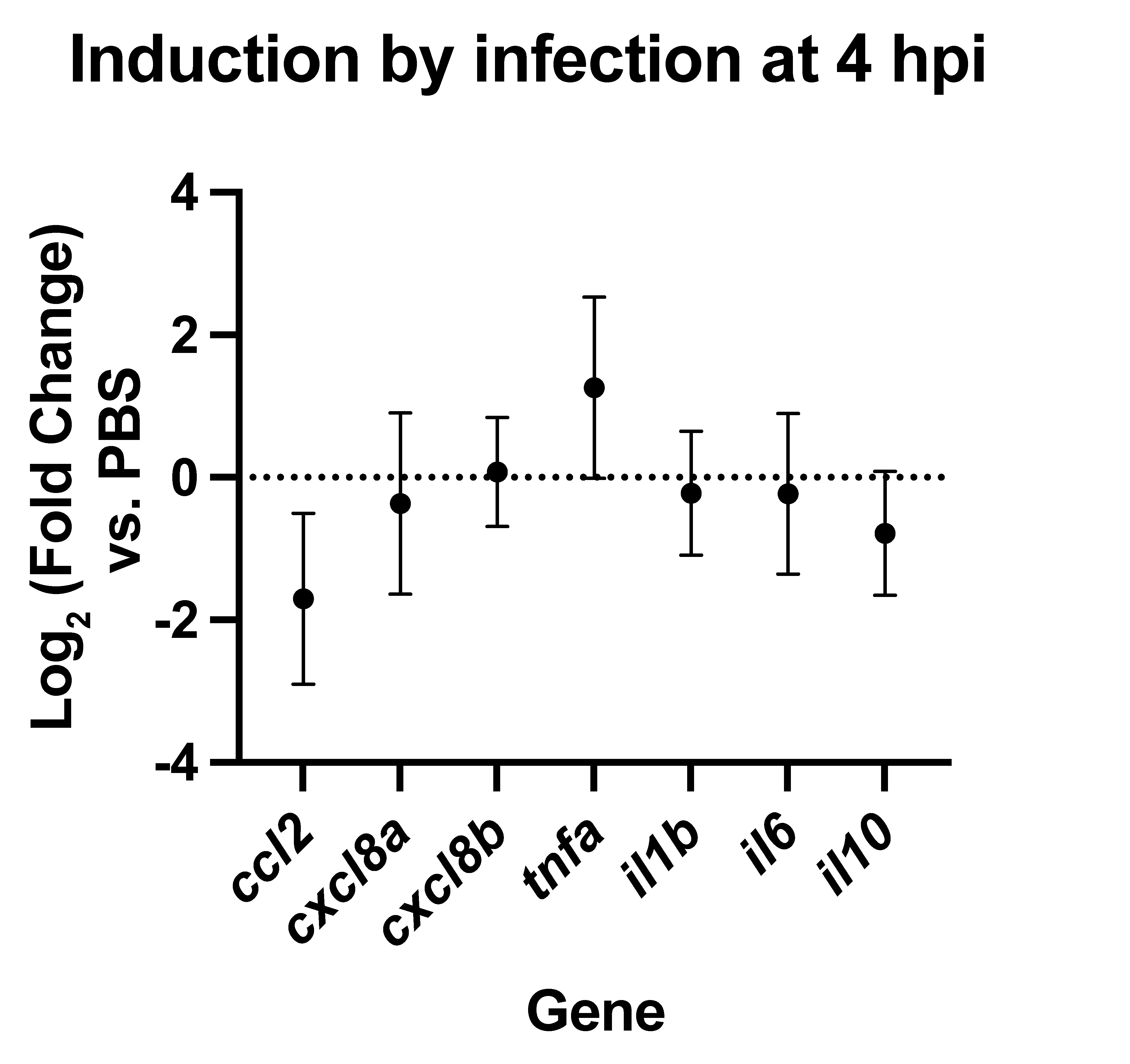

Supplement: Fig. S4 — Expression of inflammatory genes early during C. albicans infection. [file mbio.00529-25-s0005.tif]

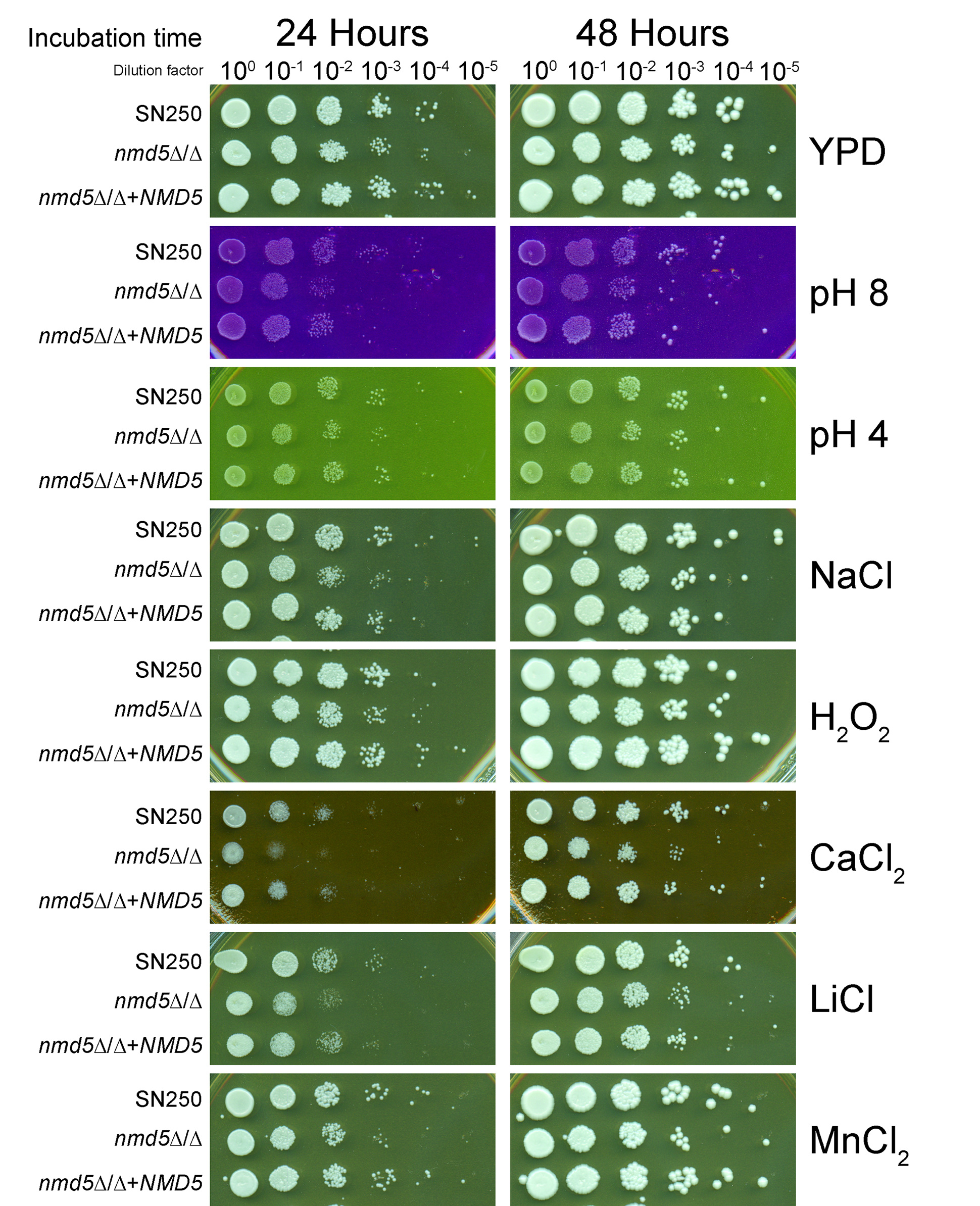

Supplement: Fig. S5 — nmd5∆/∆ is not more susceptible to cell stressors. [file mbio.00529-25-s0006.tif]

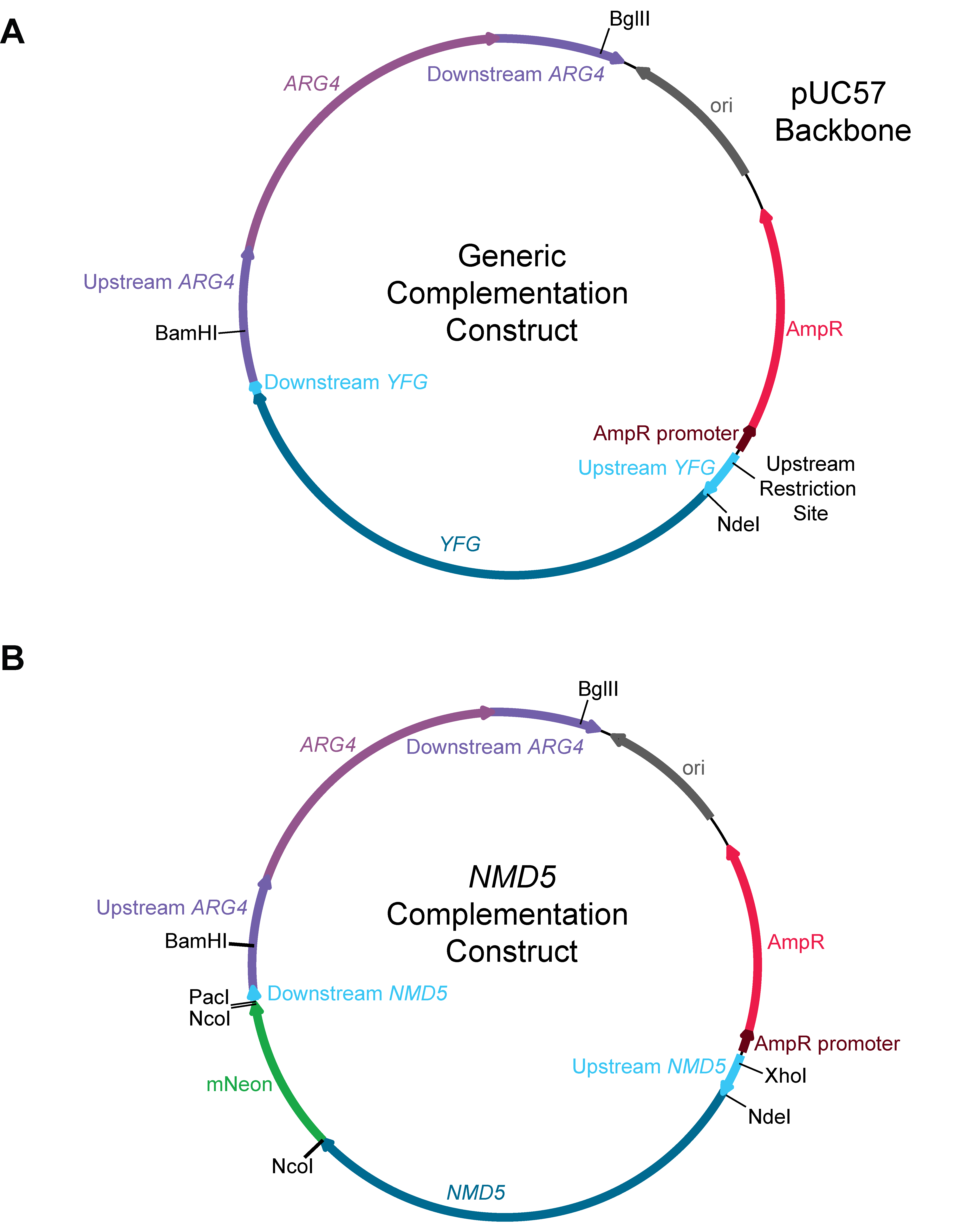

Supplement: Fig. S6 — Complementation constructs. [file mbio.00529-25-s0007.tif]
