# Supplementary material for: Forward genetic screen in zebrafish identifies new fungal regulators that limit host-protective Candida-innate immune interaction
Source: mBio. 2025 Apr 2;16(5):e00529-25. doi: 10.1128/mbio.00529-25 (PMC12077120; doi:10.1128/mbio.00529-25)
Supplement: Supplemental Legends and Captions — Legends for Fig. S1–S6 and captions for Tables S1–S5. [file mbio.00529-25-s0008.docx]

**Supplementary Figure Legends and Table Captions**

**Fig. S1. Complementation partially restores *in vitro* phenotypes of *brg1*∆/∆, *pep8*∆/∆, *cek1*∆/∆ and *rim101*∆/∆ mutants.** Growth of SN250 (**A-C)**, *brg1*∆/∆, *brg1*∆/∆+*BRG1* (**A),** *pep8*∆/∆, *pep8*∆/∆+*PEP8* (**B),** *cek1*∆/∆, and *cek1*∆/∆+*CEK1* (**C)** on Spider agar after 7 and 14 days at 30°C. **D)** Growth of SN250, *rim101*∆/∆, and *rim101*∆/∆+*RIM101* in M199 pH 4 or pH 8 after 4 hours at 37°C. Scale bar is 20 µm.

**Fig. S2. Complementation did not restore virulence of *cht2*∆/∆, *orf19.5547*∆/∆, or *rbt1^968-2166^*∆/∆. A)** Kaplan-Meier survival curve of fish injected with PBS (mock, n=23), SN250 (WT, n=41), *cht2*∆/∆ (n=31), or *cht2*∆/∆+*CHT2* (n=44). Data pooled from 2 experiments. **B)** Kaplan-Meier survival curve of fish injected with PBS (mock, n=10), SN250 (WT, n=21), *orf19.5547*∆/∆ (n=16), or *orf19.5547*∆/∆+*ORF19.5547* (n=19). Data from 1 experiment. **C)** Kaplan-Meier survival curve of fish injected with PBS (mock, n=58), SN250 (WT, n=84), *rbt1^968-2166^*∆/∆ (n=90), or *rbt1^968-2166^*∆/∆+*RBT1* (n=105). Data pooled from 5 experiments. **D)** Kaplan-Meier survival curve of fish injected with PBS (mock n=35), SN250 (WT, n=60), *rbt1^968-2166^*∆/∆ (n=52), or *rbt1*∆/∆ (n=60). Data pooled from 3 experiments.

**Fig. S3. Some mutants show fewer elongated cells in the zebrafish hindbrain at 4-6 hours post infection. A)** Representative images of SN250 and *brg1*∆/∆ infected fish showing yeast (arrow heads) and elongated cells (arrows) in the zebrafish hindbrain at 4-6 hours post infection. Scalebars are 50 µm. **B)** Plot showing the percent of elongated cells for each mutant with the control SN250 for the same experiments. The number of yeast and elongated cells was counted manually in at least 3 independent experiments for each mutant, with approximately 25 fish per strain imaged. There are separate SN250 columns for each set of experiments, as the mutant was compared to wildtype in the same experiments. Shading indicates the Groups I-IV, based on similar fungal-immune interaction phenotypes (Table 1). Means and 95% confidence intervals are plotted. Hedges bias-corrected effect sizes and significance was determined for each mutant. * indicates p<0.05, *** indicates p<0.001, # indicates a moderate effect, ## indicates a large effect.

**Fig. S4. Expression of inflammatory genes early during *C. albicans* infection.** Zebrafish larvae at the prim25 stage were infected with 10-25 *C. albicans* cells of the wildtype SN250 strain. At 4-6 hours post-infection, they were euthanized, RNA was purified, and qPCR was conducted to determine the change in gene expression relative to the mock-infected controls at the same time point. There was no significant induction of any of these inflammatory genes at this timepoint, although there was a slight reduction in *ccl2* expression. Shown are averages and 95% confidence intervals for seven biologically independent experiments. Significant changes were determined by comparing the 95% confidence intervals; none were significantly up-regulated.

**Figure S5. *nmd5*∆/∆ is not more susceptible to cell stressors.** Growth of SN250, *nmd5*∆/∆, and *nmd5*∆/∆+*NMD5* on YPD, M199 pH 8, M199 pH 4, YPD + 400 mM NaCl, YPD + 1.5 mM H_2_O_2_, YPD + 400 mM CaCl_2_, YPD + 150 mM LiCl, and 6 mM MnCl_2_. 3x10^7^ cells from the overnight culture was inoculated into 5ml fresh YPD and incubated on roller drum for 4 hours. After 4 hours 10-fold serial dilutions 3μl of the dilutions was spotted onto plates. Plates were incubated at 30°C for 48 hours and imaged after 24- and 48-hours incubation.

**Fig. S6. Complementation Constructs** **A)** Plasmid showing the design of the construct for complementation of mutant strains. All plasmids contained a BglII cut site downstream of *ARG4*, a BamHI cutsite upstream of *ARG4*, an NdeI cutsite at the ORF start site, and another restriction cut site in the complementary upstream region of the gene of interest. The upstream restriction site and the BglII restriction site were used to excise the fragment for complementation. **B)** Plasmid showing the design of the construct for complementation of *nmd5***∆/∆**. The *NMD5* complementation construct contains mNeon to enable screening of transformants for fluorescence to assess functional complementation. The XhoI and BglII restriction sites were used to excise the fragment for complementation. Sequences of ORFs with upstream and downstream regions used in complementation constructs is provided in Table S5.

**Table S1. Full list of *C. albicans* strains used in this study.** All mutants tested in this study and which stages of testing they passed.

**Table S2. Phagocytosis efficiency for Calcofluor White-labeled mutant *C. albicans* infections.** Summary statistics of the immune response to infection for all mutant *C. albicans* infections imaged, as shown in Fig. 1E.

**Table S3. Immune response to mutant *C. albicans* infections.** Summary statistics of the immune response to infection for all mutant *C. albicans* infections imaged in double transgenic fish, as shown in Fig. 4 and Fig. 5.

**Table S4. Comparison of mutant virulence in zebrafish hindbrain infection versus mouse tail vein infection.** Breakdown of virulence phenotypes for all mutants included in this screen.

**Table S5. Complementation Construct Sequences.** Sequences used for complementation for each mutant, as well as *C. dubliniensis ARG4* used in each of the complementation constructs. Complementation constructs were constructed by inserting these sequences into the pUC57 vector.
